# Nature versus Number: Monocytes in Cardiovascular Disease

**DOI:** 10.3390/ijms22179119

**Published:** 2021-08-24

**Authors:** Helen Williams, Corinne D. Mack, Stephen C. H. Li, John P. Fletcher, Heather J. Medbury

**Affiliations:** 1 Vascular Biology Research Centre, Department of Surgery, Westmead Hospital, Westmead, Sydney, NSW 2145, Australia; Helen.williams@sydney.edu.au (H.W.); cmac3879@uni.sydney.edu.au (C.D.M.); john.fletcher@sydney.edu.au (J.P.F.); 2 Westmead Clinical School, The University of Sydney, Westmead, Sydney, NSW 2145, Australia; 3 Chemical Pathology, NSW Health Pathology, Westmead Hospital and Institute of Clinical Pathology and Medical Research, Westmead, Sydney, NSW 2145, Australia; Stephen.Li@health.nsw.gov.au; 4 Blacktown/Mt Druitt Clinical School, Blacktown Hospital, Western Sydney University, Blacktown, NSW 2148, Australia

**Keywords:** monocytes, cardiovascular disease, dyslipidemia, atherosclerosis, inflammation, macrophage

## Abstract

Monocytes play a key role in cardiovascular disease (CVD) as their influx into the vessel wall is necessary for the development of an atherosclerotic plaque. Monocytes are, however, heterogeneous differentiating from classical monocytes through the intermediate subset to the nonclassical subset. While it is recognized that the percentage of intermediate and nonclassical monocytes are higher in individuals with CVD, accompanying changes in inflammatory markers suggest a functional impact on disease development that goes beyond the increased proportion of these ‘inflammatory’ monocyte subsets. Furthermore, emerging evidence indicates that changes in monocyte proportion and function arise in dyslipidemia, with lipid lowering medication having some effect on reversing these changes. This review explores the nature and number of monocyte subsets in CVD addressing what they are, when they arise, the effect of lipid lowering treatment, and the possible implications for plaque development. Understanding these associations will deepen our understanding of the clinical significance of monocytes in CVD.

## 1. Introduction

Cardiovascular disease (CVD) is a leading cause of death and disability worldwide [1]. The main etiological factor for CVD is atherosclerosis which principally affects the coronary, as well as the cerebrovascular and peripheral circulation, with risk of heart attack, stroke and limb loss. The clinical presentations of coronary artery disease (CAD) include unstable and stable angina pectoris, myocardial infarction (MI), cardiac arrhythmia and congestive cardiac failure, but CAD may also be asymptomatic and only detected on specific investigations [2]. Atherosclerosis in the cerebrovascular circulation is commonly localized to the carotid artery bifurcation region in the neck [3]. Carotid artery disease can be asymptomatic, but progressive and unstable plaque carries a significant risk of stroke. In peripheral arterial disease (PAD), atherosclerosis may be localized, but it more frequently tends to be diffuse [4], often with long segment occlusions. Arteries predominantly affected are in the aorto-iliac (supra-inguinal) and femoropopliteal (infra-inguinal) segments. Renal arteries to the kidneys and mesenteric arteries supplying the gastro-intestinal tract are much less frequently affected by atherosclerosis. However, renal artery disease can be a factor in hypertension and chronic kidney disease. Regardless of location, atherosclerosis can cause flow-limiting narrowing. More importantly, the erosion or rupture of unstable plaques can cause atherothrombotic material to occlude arteries, and results in an ischemic event in the coronary, cerebrovascular or peripheral circulation.

Risk factors for CVD are broadly considered either non-modifiable (e.g., age, sex, family history of premature CVD) or modifiable (e.g., hypercholesterolemia, hypertension, tobacco smoking, overweight or obesity, insulin resistance or diabetes, lack of physical activity). Addressing these modifiable risk factors could prevent or delay the atherosclerotic process [5]. Among risk factors, dyslipidemias are considered key contributors to the development of atherosclerotic plaque. Dyslipidemia has many clinical presentations, and some of those considered important for CVD risk include high levels of low density lipoprotein (LDL) or its protein component apolipoprotein (Apo)B, low levels of high density lipoprotein (HDL) or its protein component ApoA1, and, more recently, high levels of lipoprotein(a) [Lp(a)] [6]. While CVD risk reduction can be achieved by returning these altered lipids to target levels, this does not affect other facets of risk. Understanding the other risk factors present in a patient allow clinicians to assess absolute CVD risk and formulate suitable therapeutic approaches. 

Assessment of absolute CVD risk would be incomplete without considering inflammation. Levels of high sensitivity C-reactive protein (hs-CRP) are used clinically as a marker of inflammation and can reflect the degree of vascular inflammation, which can be induced by dyslipidemia. Within people with the same levels of blood cholesterol, differences in hs-CRP have been implicated as a contributing factor to CVD outcomes [7]. Furthermore, targeting key inflammatory pathways, such as in the CANTOS trial, has been shown to reduce cardiovascular risk, independent of lipid lowering [8].

Of the immune cells involved in inflammatory pathways in CVD, monocytes and macrophages have become a focal point. Macrophages are a predominant cellular component of the atherosclerotic plaque and are a heterogeneous population of different functional phenotypes [9], which have different implications for plaque stability or instability [10]. Inflammatory macrophages, distinguished as the ‘M1’ subset, produce higher levels of inflammatory mediators including cytokines and matrix metalloproteinases which promote degradation of the fibrous cap, destabilizing the plaque structure [10,11]. Clinically, increased proportions of inflammatory M1 macrophages are found in the caps of unstable plaques [12]. Conversely, the ‘M2’ subset of macrophages demonstrate wound-healing functions including increased secretion of anti-inflammatory cytokines and production of collagen in carotid plaques, supporting plaque stabilization [12,13]. 

Infiltrating monocytes are a key source of plaque macrophages. They arise from precursors in bone marrow and are released into the circulation, where they constitute 3–8% of white blood cells [14]. Mouse studies have shown that blocking monocyte influx into the plaque can reduce development of atherosclerosis [15]. In humans, high levels of monocytes are seen with CVD development, and their numbers are emerging as an indicator of CVD severity. Considering monocyte-derived macrophages play a pivotal role in the inflammatory processes that determine plaque progression and instability, alterations in the monocyte population may influence the inflammatory functions of macrophages and thus promote the progression of an unstable atherosclerotic plaque. Indeed, monocyte phenotypes and functions are emerging as indicators of inflammation in CVD, and furthermore, disease severity. To understand the clinical significance of monocytes in CVD, it is important to delineate both the impact of changes in monocyte *number* and functional *nature*. The monocyte alterations of relevance to CVD, and dyslipidemia, will be the focus of this review, with a discussion of more recent insight into how these changes may be promoting plaque progression. 

## 2. Monocyte Subset Proportions in CVD

**Figure 1:**
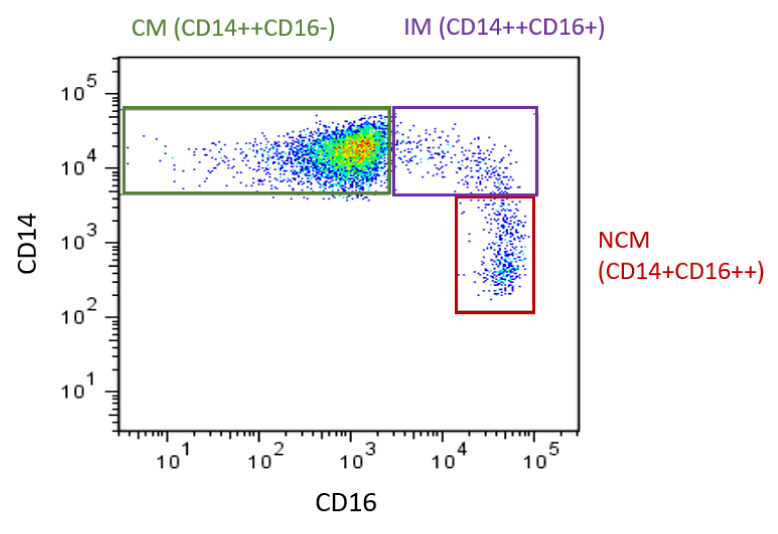
Monocyte subsets in humans. Flow cytometry plot produced by the authors displaying monocyte subsets from a healthy control. Monocytes are defined using CD14 and CD16. CM: classical monocytes, IM: intermediate monocytes, NCM: nonclassical monocytes.

The heterogeneity of monocytes has been increasingly recognized, with this considered to have implications for CVD. Initially, monocytes were classified into two main subsets based on the expression of specific surface markers: both express CD14 but they differ in CD16 expression. These two subsets were referred to as CD16- and CD16+. Of these, CD16+ monocyte counts are elevated in CVD [16]. In 2010, the definition of monocyte subsets was expanded to three subsets. Still based on expression of CD14 and CD16 [17], the three recognized subsets are CD14++CD16- (classical, Mon 1), CD14++CD16+ (intermediate, Mon2), and CD14+CD16++ (nonclassical, Mon3) (Figure 1) [14,17]. Classical monocytes make up ~85% of circulating monocytes, with intermediate and nonclassical being ~5% and 10%, respectively. The use of flow cytometry to identify and enumerate these subsets is widespread. However, as specific methods (in particular the gating steps used to identify the subsets [18]) vary between groups, comparisons between studies can be difficult, as different gating yields different subset percentages. It has been shown that inclusion of a pan monocyte marker, such as CD86 or HLADR in the staining panel, is essential to ensure non-monocyte cells are excluded during the gating steps [19]. Additional monocyte surface markers have also been proposed to allow better separation of the intermediate and nonclassical subsets, such as their respective positive and negative expression of the chemokine receptor CCR2 [20]. Recently, it has been proposed that more than three subsets exist [21,22], which is unsurprising considering monocytes exist on a continuum (of expression of CD14 and CD16: as seen in Figure 1) rather than in discrete populations and that even within each subset, the expression of markers (such as adhesion molecules) is heterogeneous [23]. While classification into three subsets may be simplistic, it has been found to be relevant in studies of monocyte associations with CVD.

In CVD, the proportion of intermediate and/or nonclassical monocytes are frequently reported to be elevated (Table 1). There also appears to be further increases in these subsets with increased severity of disease. Patients with atherosclerosis in multiple vessels had significantly more CD16+ (intermediate + nonclassical) monocytes than those with single vessel disease [24]. This relationship was also seen when patients were divided by proportion of monocyte subsets, with those below median for classicals or above median for intermediate or nonclassicals having a higher occurrence of multivessel disease [25]. In PAD patients, the presence of ischemic ulcer (an indicator of greater disease severity) is associated with decreases in classical monocyte proportion and increases in intermediate monocyte proportion [26]. Similarly, high counts of intermediate and nonclassical monocytes have been observed in unstable angina compared to stable coronary heart disease (CHD) [27]. Lastly, when CAD was graded as obstructive, non-obstructive, or minimal, the obstructive and non-obstructive groups had higher intermediate monocytes than the group with minimal CAD [28].

Occurrence of clinical events is also linked to subset proportion changes. In stroke, patients had higher intermediate monocytes than controls [29]. Furthermore, elevations in monocyte subsets are associated with complications, with high levels of classical monocytes predictive of poor outcome after a stroke [29] and higher levels of intermediate monocytes seen in patients presenting for coronary angiography who subsequently had a cardiovascular event (death, stroke or acute MI) [30].

Could monocyte proportion changes influence plaque stability? There is evidence to suggest they can. In controls, intermediate monocyte count had a strong association with plaque severity [40]. In patients with HIV, a baseline elevation in nonclassical monocytes was linked to coronary artery calcification [41]. Similarly, correcting altered proportions of monocytes could help stabilize plaques, as treatment of unstable angina pectoris patients resulted in an increase in fibrous cap thickness, and this was associated with a reduction in CD16+ (intermediate + nonclassical) monocytes. These studies show monocyte proportion changes have a direct association with the plaque and could influence its stability. 

It is clear that despite variations in proportions presented by different groups, there is a consistent pattern of elevated intermediate and/or nonclassical monocytes in CVD, and these are linked to severity of disease and its clinical manifestations. To understand possible mechanisms linking this elevation in proportions to CVD development and severity, consideration of how the functions of these monocyte subsets differ is important.

## 3. Monocyte Functional Changes in CVD

The elevation in intermediate monocytes in CVD is considered to promote disease progression, through increased inflammation. Intermediate monocytes (compared to the other subsets) have been implicated as major producers of inflammatory cytokines, including Interleukin (IL)-1β, IL-6 and Tumor necrosis factor (TNF)α in response to lipopolysaccharide (LPS: an outer membrane component of bacteria) [42]. Similarly, the monocyte subsets have been shown to have different potential for adhesion and migration [23,43], therefore proportional changes may further impact atherogenesis through altered monocyte entry to the plaque. 

There are two caveats of assuming that proportional changes indicate a pro-atherogenic monocyte profile. Firstly, it hinges upon the accurate identification of subsets and secondly, the validity of the functional differences between the subsets may be uncertain due to varying experimental approaches. As mentioned in the previous section, there are differences in the flow cytometry gating approaches used to identify monocyte subsets by different research groups. Such inconsistencies between studies limit comparisons between them. In addition, inaccurate identification of the subsets (through flow analysis gating issues) could cause downstream errors in assigning functions to these subsets. As suitable identification of these subsets has been established [19,44], future studies should be able to be more consistent in identifying the three monocyte subsets. The second concern regarding functional assessment of subsets is due to functions from in vitro studies being assigned to these same cells in vivo. For example, cytokine release is often measured after in vitro stimulation with LPS, which does not replicate the microenvironment monocytes are exposed to in the blood, and thus may not entirely reflect the release of these cytokines in vivo.

While changes in monocyte subset proportions are taken as broadly indicative of overall functional changes in circulating monocytes, this is not the full picture. It is important to consider whether other monocyte changes, either independent of, or in addition to, subset proportions, also occur in CVD. Firstly, considering monocyte entry into the plaque, enhanced adhesion or migration capacity of monocytes could hasten plaque progression. Indeed, monocytes from CAD patients have increased adherence to endothelial cells [45]. Increased adherence could be attributed to elevations in monocyte adhesion molecules, such as the β2-integrin family (CD11a, CD11b, or CD11c complexed with CD18). In CVD, elevations in adhesion markers (CD11b/CD18) have been reported on monocytes [46] (Table 2). That CD18 is increased with greater CVD severity indicates further enhanced adhesion capacity as CVD progresses [28]. Enhanced ability to adhere does not necessarily equate to increased migration into the plaque, but elevated monocyte chemokine receptors indicate enhanced migration capacity in CVD. The increased level of chemokine receptors (CCR2 and CX3CR1) on monocytes in CAD [31] could contribute to enhanced monocyte accumulation in atherosclerotic plaques. Again, advancing severity may see a further increase, as higher CX3CR1 was seen with advancing stenosis [28].

Monocyte inflammatory alterations have also received attention. When stimulated under M1 macrophage polarizing conditions (LPS/IFNγ), monocytes from people with CAD showed a greater gene expression of inflammatory cytokines (IL-6 and IL-1β) compared to controls [35], though another study found little difference in cytokine release between CAD and controls [20] (Table 2). The pathway leading to inflammatory cytokine release is also altered in CVD, with high levels of Toll-like receptor (TLR) 4, an LPS receptor, correlated with serum levels of TNFα [48]. Further activation of this inflammatory pathway is likely to occur with clinical events, as an elevation in TLR4 has been seen in both ST-elevation myocardial infarction (STEMI) and Non-ST-elevation myocardial infarction (NSTEMI) compared to CAD [49]. Apart from direct measurement of cytokine release or factors in this pathway, other indicators of inflammation have been shown to be elevated in CVD. Circulating monocytes in people with CVD resemble inflammatory macrophages (M1), owing to their high CD86/CD163 surface marker expression ratio relative to controls [37]. The CD86/CD163 ratio was associated with LPS-induced expression of IL-1β, indicating this resemblance is accompanied by functional impact [37]. Of interest, resemblance of monocytes to M1 macrophages has also been seen in people with risk factors for CVD such as diabetes and obesity [50,51]. Occurrence of a clinical event has also been linked to inflammatory alterations in monocytes, with patients who had experienced STEMI and NSTEMI having lower levels of the “M2” markers CD163 and CD206 on their monocyte-derived macrophages [39].

Thus, in addition to the implied functional alterations arising from monocyte subset proportion changes, altered adhesion, migration and inflammatory potential of monocytes has been seen in CVD, and are likely to promote disease progression. To understand what may cause these changes, examining alterations in light of risk factors could be beneficial. 

## 4. Monocyte Count/Percentage Changes Relative to Lipids

While monocyte subset proportions are altered in CVD, it is likely that lipid levels are partly responsible for the altered proportions. High levels of atherogenic lipids, such as Lp(a) and small dense (sd)LDL are associated with increased intermediate monocytes [52,53], whereas HDL-C and ApoA1 levels are inversely associated with intermediate monocyte counts [54,55] (Table 3). HDL can impact monocyte development, as it has been shown in murine models that HDL suppresses stem cell proliferation [56,57] and reduces monocytosis [57]. Clinically, there is an inverse correlation between HDL-C level and the level of expression of CD16 on monocytes [58]. The change in CD16 expression would explain the increased proportion of intermediate and nonclassical monocytes in dyslipidemia (Table 3) and in CVD (Table 1) as, by definition, upregulation of CD16 expression would see monocytes no longer classed as ‘classical’ but deemed to be intermediate (or nonclassical) monocytes [58].

## 5. Monocyte Functional Changes Associated with Lipid Levels

**Figure 2:**
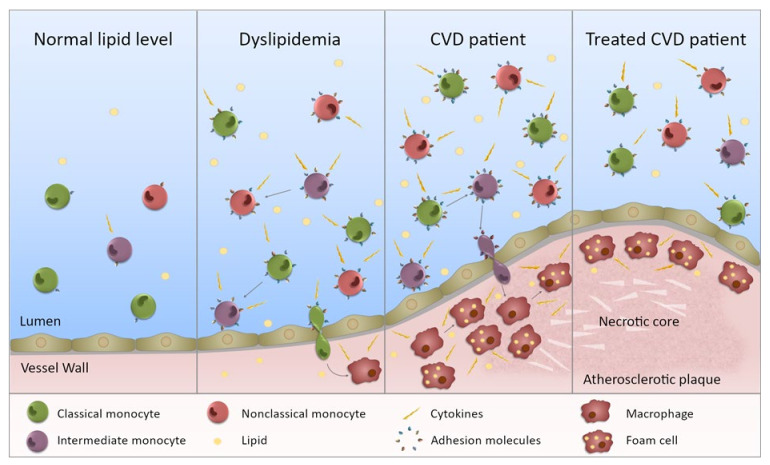
Monocytes in dyslipidemia and CVD. This figure illustrates the state of monocytes in CVD. **Normal lipid levels:** in individuals with normal lipid levels, inflammation is low and the different monocyte subsets preferentially express specific adhesion molecules and chemokine receptors. **Dyslipidemia:** in individuals with dyslipidemia, the monocyte subset proportion is altered with increased intermediate and nonclassical monocytes, presumably through maturation of monocytes from one subset to the next. In addition, all subsets are more inflammatory (not just one) with increased ability to produce inflammatory cytokines. The subsets also express an increased level, and range of, adhesion molecules (and chemokine receptors) giving them a greater potential to bind to the endothelium. Entering the vessel wall, they become macrophages and upon lipid uptake, transform into foam cells. **CVD patient:** in CVD patients, the inflammatory state of the monocytes is further exacerbated and subset proportions further perturbed. Monocytes are more able to enter the vessel wall and to adopt an inflammatory macrophage phenotype once there. The chronic inflammatory state will contribute to plaque development. **Treated CVD patient:** upon treatment, the monocyte subsets become less inflammatory, but they do not necessarily return to levels equivalent to controls. Any lipid-associated functional changes of monocyte derived macrophages in the atherosclerotic plaque that have been induced by trained immunity may persist. Thus, plaque progression could continue despite a drop in lipid levels.

Aside from changes in monocyte subset proportions, monocyte inflammatory profile and migration is also associated with lipid levels (Table 4 and illustrated in Figure 2). Firstly, monocytes from hyperlipidemic patients display increased cytokine production (post LPS stimulation) [61] and adhesion to endothelial cells [61,62] compared with controls. Secondly, even for generally healthy controls, monocytes are skewed towards an atherogenic state in individuals with a perturbed lipid profile [23,37,42]. Thirdly, high fat feeding increases monocyte adhesion and chemotaxis [63,64]. These all point to there being an immunopathology associated with dyslipidemia, one that begins in generally healthy individuals with perturbed lipids. Dyslipidemia is highly prevalent in adults [65,66]. Monocyte adoption of a pro-atherogenic phenotype is likely occurring largely undetected in many of these individuals, as dyslipidemia is generally asymptomatic [67]. Furthermore, these atherogenic monocytes could be contributing to plaque development, unabated in many, as treatment for dyslipidemia is recommended relative to risk of a cardiovascular event [68] and as such, younger dyslipidemic adults are usually not pharmacologically treated. The increase in monocyte CD11c expression and adherence to VCAM-1 (an adhesion molecule found on endothelial cells) observed after high fat feeding [63] would further promote monocyte migration into the vessel wall. Though of note, the increase in CD11c expression was transient, with its return to baseline matching the change in triglyceride levels [63]. With dyslipidemia being a major risk factor for CVD, the altered monocyte phenotype in dyslipidemia may explain, in part, the increased inflammatory profile of monocytes in CVD (Table 2).

Of note, the skewing of monocytes to an atherogenic state in generally healthy individuals with a perturbed lipid profile was evident for all three monocyte subsets [23,42]. Though there were still notable differences between the subsets, these were overshadowed by differences between individuals, such that in some individuals, all three monocyte subsets produced inflammatory cytokines or expressed recruitment markers at a higher level than in other individuals. This suggests that functional distinctions between the monocyte populations are blurred in dyslipidemia, such that the whole monocyte population becomes increasingly pro-atherogenic. The inflammatory state being individual-specific, more so than subset-dependent, explains the finding (that was surprising at the time) that atherosclerosis regression in mice arose from recruitment of classical (Ly6C^hi^) monocytes that adopted an M2 macrophage phenotype in plaque, even though Ly6C^hi^ monocytes were generally considered to be the ‘inflammatory’ subset [71]. Considering these findings, while monocyte subset count/percentage may be associated with disease state, selective targeting of a specific population to improve clinical outcomes is unlikely to be effective.

The phenotype that monocytes acquire in generally healthy individuals, dyslipidemia or CVD (Table 2, Table 4 and Table 5) is lipid-specific with, for example, TLR2 increased relative to total and LDL cholesterol [42], while most of the adhesion markers examined displayed an inverse relationship with ApoA1 [23]. The inverse correlation of the recruitment markers examined with ApoA1 [23] suggests that monocytes have an increased potential to extravasate when ApoA1 is low. While this is consistent with the findings that high levels of ApoA1 decrease both chemokine receptor levels and monocyte migration [72], it is also recognized that high ApoA1 can, through cholesterol depletion of lipid rafts (lipid-stabilized membrane microdomains in which cell receptors cluster), dampen the signaling pathways required for the reorganization of the actin cytoskeleton that is needed for cell migration [73]. That the level of several recruitment markers was increased on the three monocyte subsets in individuals with low ApoA1 [23] suggests that all subsets may be able to migrate into the vessel wall (or other sites of injury) and do so using a wider repertoire of adhesion molecules than for individuals with normal ApoA1 levels. Indeed, murine models of atherosclerosis are associated with the influx of both classical (Ly6C^hi^) and nonclassical (Ly6C^low^) monocytes, not just one subset [74].

Interestingly, in dyslipidemic patients, monocyte TNFα release correlated with total cholesterol and ApoB, and IL-1β release positively correlated with total cholesterol [79], while in contrast in healthy controls, IL-1β production correlated inversely with HDL-C and ApoA1 [42]. The Okopien study [79] was much larger and, notably, a greater proportion of the individuals had elevated cholesterol than in the smaller Patel study [42], which may have enabled more associations with cholesterol to be seen. However, also, ApoA1 was not measured in the Okopien study [79]. As IL-1β production is regulated by lipid rafts, the integrity of which is dependent on cholesterol content [86], then both cholesterol overall and HDL may impact cytokine production through this mechanism. However, as Okopien [79] did not find any correlation between the drop in cytokine levels and extent of lipid lowering, and also found that cytokine levels dropped in those for whom the decrease in LDL-C was slight, it was suggested that the effect of statin treatment was not lipid related but involved other anti-inflammatory pathways. Similarly, while it was found that TLR4 expression was related to lipid levels, the reduction in TLR4 upon statin treatment of ex vivo monocytes was independent of the cholesterol lowering effect of statins [69]. Fibrates were also shown to decrease cytokine production by monocytes of individuals with dyslipidemia [76,77], an effect which is also thought to be unrelated to the lipid lowering effect of fibrates.

Though the drop in monocyte cytokine production (upon lipid lowering) was sometimes to the level of that of the controls [79,84], this was not always the case [82,85] (Table 5). Moreover, in an analysis of monocytes from FH patients’ post-statin treatment [85], Bekkering found that despite there being a large drop in LDL-C level, the monocyte RNA expression profile did not change to a great extent upon statin treatment. Patients’ monocytes were still enriched for metabolic and inflammatory pathways, with evidence of epigenetic modification of the proinflammatory *TNFA* gene [85].

## 6. Impact of Functional Changes

These alterations to monocyte function and phenotype, outlined above in both CVD and in dyslipidemia, are likely to have critical implications for atherosclerosis progression (Figure 2), due to the fact that monocytes are a source of plaque macrophages [10,11,12,13]. Previously, the phenotype of plaque macrophages was thought to be solely determined by factors in the plaque microenvironment, such as cytokines [87,88]. However, there is now growing evidence that factors in the circulation can cause functional changes in monocytes that impact the functional state of the differentiated monocyte-derived macrophages [89,90]. Therefore, inflammatory changes to circulating monocytes in CVD or dyslipidemia may persist through differentiation, resulting in an inflammatory macrophage phenotype promoting plaque destabilization. Indeed, there is evidence in CVD of a heightened inflammatory monocyte phenotype persisting in monocyte-derived macrophages [35]. Not only do monocytes isolated from CAD patients show a higher inflammatory cytokine response to LPS stimulation (IL-1β, IL-6, TNFα) compared to controls (Table 2), but this heightened inflammatory response remains present in macrophages derived from their in vitro culture [35]. Further evidence of skewed monocyte–macrophage differentiation is found in the context of obesity, where the peripheral blood mononuclear cells of obese individuals produced higher levels of inflammatory cytokines (IL-1β, TNFα, IFNγ) and had a restricted ability be polarized to an M2 macrophage phenotype (indicated by lower expression of M2 markers (CD163, CD200R, CD204, TGFβ), compared to healthy controls [91].

As it has been shown that monocyte functional changes associate with dyslipidemia, exposure to perturbed lipid levels may subsequently result in the skewed differentiation to inflammatory plaque macrophages. When exposed to LDL or its oxidized form (oxLDL) during culture, M1 monocyte-derived-macrophages display an enhanced inflammatory cytokine response (IL-1β, IL-6, TNFα); while in M2 monocyte-derived-macrophages, LDL exposure reduces M2 surface marker expression (CD206, CD200R) and anti-inflammatory cytokine production (TGFβ, IL-10) [92]. Conversely, the presence of HDL during macrophage culture inhibits polarization to an M1 phenotype (reducing M1-associated expression of CD192, CD64, TNFα, IL-6, MCP-1 and reactive oxygen species generation); while M2 polarization is unaffected [93]. As with monocytes, the effect of HDL on macrophages was due to its interfering impact on the membrane lipid rafts which are involved in activating inflammatory gene expression [93]. Specifically, this was mediated through the ApoA1 component of HDL inducing cholesterol efflux from the cells, subsequently reducing the cholesterol content of these lipid rafts [94].

There is also evidence that some inflammatory changes in monocyte–macrophage differentiation associated with CVD and dyslipidemia may be the result of a more stable cellular reprogramming, which would persistently promote vascular inflammation despite lipid-lowering treatment. *Trained immunity* is a phenomenon whereby monocytes form a metabolic and epigenetic memory that enables them to mount a heightened inflammatory response to secondary stimuli, even after the initial stimuli are removed [90]. This effect is indeed evident in atherosclerosis, as monocytes isolated from patients with symptomatic coronary atherosclerosis demonstrate an increased inflammatory cytokine response to LPS stimulation (TNFα, IL-6, IL-1β, IL-8, MCP-1). This response was associated with the mechanisms of trained immunity, specifically increased expression of glycolytic enzymes and epigenetic modifications to inflammatory genes [95]. As in vitro studies demonstrate that a trained response persists after macrophage differentiation [90], then it can be expected that these changes will result in inflammatory macrophage differentiation in the plaque. However, this effect was not observed in patients with asymptomatic atherosclerosis [95], suggesting that, clinically, trained immunity may only be evident in progressed disease.

Furthermore, the finding of Bekkering (mentioned above) that despite significant LDL lowering, statin treatment in FH patients was unable to reverse inflammatory monocyte changes, provides evidence of trained immunity in dyslipidemia [85]. While statins could reverse inflammatory changes in some cases of dyslipidemia (Table 5), they were unable to reverse the increased expression of metabolic genes and epigenetic modifications to inflammatory genes that may have been induced by the extensive duration of elevated lipids in FH [85]. Such functional reprogramming suggests that despite lipid-lowering by statin treatment, any lipid-associated functional changes to monocytes in CVD or dyslipidemia that are induced via trained immunity will persist and likely drive their differentiation to an inflammatory macrophage phenotype in the plaque.

While it has been demonstrated that native LDL does not induce a trained immune response, oxLDL is established as a key stimulus for the induction of trained immunity in monocyte–macrophage differentiation [96]. Monocytes treated with oxLDL, washed, cultured into macrophages and restimulated with LPS show increased inflammatory cytokine and chemokine production and foam cell formation. This is dependent on increased glycolysis and associated with epigenetic modifications to the promoters of inflammatory genes [96,97]. Lp(a), both isolated and in serum from an individual with elevated levels, has also been shown to induce trained immunity (increased TNFα and IL-6 response) through a similar in vitro protocol, whereas an increased cytokine response was not observed for LDL or HDL [98].

If trained immunity in response to certain lipids ‘locks’ monocyte differentiation to an inflammatory macrophage phenotype, then macrophages may be unable to demonstrate the wound-healing functions necessary for plaque stabilization in CVD. Normal wound healing involves a switch in macrophage phenotype from M1 to M2, however, this switch is dysfunctional in chronic wounds such as the atherosclerotic plaque [99]. While some studies suggest there is plasticity in macrophage phenotypes [11], it has been demonstrated that the metabolic changes associated with M1 macrophage polarization, many of which are also evident in trained immunity by oxLDL and in atherosclerosis [95,96,97], prevent inflammatory macrophages from being able to respond to M2 stimulus [100]. As such, inflammatory monocyte changes evident in CVD and dyslipidemia which result from trained immunity may restrict these cells from becoming wound-healing macrophages in the plaque, so that plaque instability persists, even after lipid-lowering treatments. This may contribute to the residual risk of a clinical event that CVD patients face, despite optimal therapy.

Considering the ongoing COVID-19 pandemic, a brief word on links between this condition and CVD is pertinent. A bidirectional relationship between CVD and COVID-19 has been proposed, with presence of CVD increasing risk of poor COVID-19 outcomes, and the presence of COVID-19 increasing the risk of cardiovascular outcomes [101]. Similar to in CVD, monocyte alterations could be one contributor to this relationship, as CD16+ monocytes have been reported to increase in COVID-19, along with inflammatory cytokine levels in the serum [102], and as we have shown here these types of changes could be mechanistically associated with CVD. Furthermore, COVID-19 outcomes are more severe in individuals with dyslipidemia, in particular, low HDL-C [103]. As outlined here, these individuals have a heightened cytokine response when challenged with LPS; a similar response when challenged with SARS-CoV-2 may perhaps contribute to the cytokine storm, particularly as HDL-C levels decrease even further in COVID-19 [104]. The heightened inflammatory response may be further exacerbated if also coupled with a trained immune response.

## 7. Conclusions

In investigating the question of monocyte nature versus number, it is evident that both the functional nature and distribution of subset numbers are not only altered in CVD, but may also contribute to disease progression. Assessment of monocyte numbers reveals that the proportions of the three subsets are perturbed in CVD and associate with disease severity, the occurrence and outcomes of clinical events, and plaque progression. While elevation of the intermediate and nonclassical subsets suggests that their ascribed inflammatory functions contribute to atherosclerosis, it is important to consider the broader functional changes both within the subsets and across the whole monocyte population, that occur in CVD. Of specific relevance to atherosclerosis are increased inflammation and migration.

These cellular changes are observed in association with specific lipids, particularly (low levels of) HDL-C, indicating that lipids may influence both monocyte subset proportions and function. As these functional changes in cytokine production, inflammatory surface marker expression and recruitment markers are evident in all three subsets, there is an overall skewing of monocytes to a pro-atherogenic profile in dyslipidemia. While lipid-lowering treatments demonstrate some ability to reduce these inflammatory functional changes, this is not always restored to control levels and some inflammatory functions may persist through differentiation into plaque macrophages. Persistent skewing of plaque macrophages to an inflammatory function, left untreated, will promote plaque instability. As such, both the proportional and functional changes to monocytes evident with increased CVD risk in dyslipidemia, and further exacerbated in CVD itself, are likely to contribute to the development and progression of atherosclerosis, and moreover, limit the ability of lipid lowering treatment to stabilize atherosclerotic plaques.

## Figures and Tables

**Table 1:** Monocyte proportion changes in CVD.

Reference	Patient Population	N=	Classical (CM%)	Intermediate (IM%)	Nonclassical (NCM%)	Other Outcomes
Tallone [31]	CAD (Stable)	13	**82 (↓)** *	3.6(=)	**9.2(↑)** *	Additionally reported a fourth population, “CD14-CD16+” but not significantly different between groups
	Control	14	87	3.3	5.8	
Dopheide [32]	PAD (CLI)	60	**66.2 (↓)** ***	**10.6(↑)** ***	15 (=)	
	PAD (IC)		**74.4 (↓)** *	**10.5 (↑)** ***	**23.3 (↑)** ***	
	Control	30	82	6	11.9	
Ozaki [24]	CAD (multi vessel)	51	Not stated	**25.5 (↑)** ***	Not stated	
	CAD (one vessel)	47	Not stated	**12.5(↑)** **	Not stated	
	Control	27	Not stated	8.5	Not stated	
Tapp [33]	CAD (Stable)	40	82 (=)	6.9 (=)	10.8 (=)	Additionally included data on STEMI, ↑ IM% and ↓ NCM% vs. control and CAD
	Control	40	83	6.4	10.6	
Shantsila [20]	CAD (Stable)	53	85 (=)	5.4 (=)	9.8 (=)	Proportions calculated from subset counts
	Control	50	84	5.8	9.8	
Sturhan [34]	CAD (Stable)	80	**82 (↓)** *	**13 (↑)** *	**5 (↑)** *	Additionally included data for acute MI, which showed the same changes as CAD vs. control but no difference from CAD group
	Control	34	90	7	3	
Shirai [35]	CAD		**69 (↓)** **	**20 (↑)** **	3.5(=)	
	Control		83	7	4	
Zhuang [36]	Unstable angina	48	82(=)	**10.6 (↑)** *	**6.97(↓)** *	Additionally presented data on STEMI, which showed elevated IM% and lower NCM% vs. controls (*p* < 0.05)
	Control	33	82	7.4	10.3	
Williams [37]	CVD (carotid endarterectomy and/or PAD)	31	~88(=)	~5(↑)*	~6(=)	Proportions estimated from graph
	Control	33	~90	~4	~5	
Brown [38]	Diffuse CAD	50	84.5(=)	7.2(=)	8.3 (=)	Control had CAD risk factors, as opposed to “healthy control” group
	Focal CAD	40	85.2(=)	6.2(=)	8.6(=)	
	Control	50	87.1	5.1	7.8	
Eligini [39]	CAD	90	85.8(=)	**10.5 (↑)** ***	3.7(=)	
	Control	25	85.1	7.6	4.6	

**Notes:** * *p* < 0.05 vs. controls, ** *p* < 0.01 vs. controls *** *p* < 0.001 vs. controls. Abbreviations: CLI, critical limb ischemia, IC, intermittent claudication.

**Table 2:** Monocyte functional changes in CVD.

Reference	Clinical Model	N=	Monocyte Association
Mazzone [46]	CAD	120	CD11b/CD18 was higher on patients with CAD and PAD compared to controls
	PAD	50	
	Control	200	
Kassirer [47]	Ischemic heart disease	45	CD11b/CD18 was higher on patients with ischemic heart disease than controls
	Control	66	
Tallone [31]	CAD (Stable)	13	Higher CCR2 and CX3CR1 on classical monocytes in CAD vs. control
	Control	14	
Shantsila [20]	CAD (Stable)	53	Upregulated IL-6 on CM and IM in CAD. TNFα production of LPS-stimulated monocytes over baseline was lower in CAD patients than controls
	Control	50	
Shirai [35]	CAD	7	Higher gene expression of IL-6 and IL-1β in response to LPS/Interferon(IFN)γ
	Control	7	
Williams [37]	CVD patients	31	CD163(M2) lower in CVD, CD86/CD163(M1/M2) ratio higher in CVD
	Control	33	
Chan [45]	CAD (Stable)	30	Increased NF-κb activity and iNOS expression in CAD
	Control	30	Increased monocyte adhesion to human endothelial cells

**Table 3:** Monocyte count/proportion changes relative to lipid levels.

Reference	Clinical Model	N=	Monocyte Association
Huang [54]	Healthy individuals	100	Inverse association between HDL-C and combined IM +NCM count ^1^
Rogacev [55]	Chronic kidney disease (CKD)	438	ApoA1 and HDL-C inversely correlated with IM counts
Krychtiuk [59]	Stable CAD	90	Small HDL correlated with NCM% and inversely with CM% Highest tertile of small HDL had increased NCM% and decreased CM% compared with the two lower tertiles
Krychtiuk [52]	Stable CAD	90	Elevated Lp(a) (>50 mg/dL) was associated with increased IM% OxPL/ApoB ^2^ correlated with IM%
Krychtiuk [53]	Stable CAD	90	Top tertile sdLDL had highest NCM% and lower CM% than the two lower tertiles
***Treatment model***			
Dai Perrad [60] ^3^	Hypertriglyceridemia	27	Triglyceride lowering treatment with Omega-3 fatty acids MAT9001 or EPA-EE decreased IM% and count, and increased the CM% and count. EPA-EE slightly, but significantly, decreased NCM%

**Notes:** ^1^ CD16+ cells compared to CD16- cells were investigated. Three monocyte subsets were not defined till after this publication. CD16+ cells equate to IM + NCM. ^2^ OxPL/ApoB: oxidized phospholipids on apolipoprotein B-100 containing lipoproteins. ^3^ Hypertriglyceridemia: Triglyceride between 200 and 400 mg/dL (2.26–4.52 mmol/L). MAT9001: Eicosapentaenoic acid (EPA) plus docosapentaenoic acid. EPA-EE: EPA ethyl esters.

**Table 4:** Monocyte functional changes relative to lipid levels.

Reference	Clinical Model	N=	Monocyte Association
Jongkind ^1^ [62]	Familial hypercholesterolemia	28	All four patient groups showed increased monocyte binding to cultured endothelial cells compared to the controls
	Polygenic hypercholesterolemia	10	
	Familial combined hyperlipidemia	17	
	Non familial combined Hypercholesterolemia/Hypertriglyceridemia	17	
	Healthy controls	18	
Devaraj [61]	Hyperlipidemic patients ^2^	16	Monocyte superoxide anion release, IL-1, TNF, IL-6, CD14, CD11b and adhesion to human endothelium were significantly increased in patients compared with controls
	Healthy controls	16	
Foldes [69]	Chronic heart failure	26	Monocyte TLR4 expression was inversely associated with total cholesterol, HDL, ApoA1 ^3^
	Healthy controls	13	
Fadini ^4^ [70]	Familial hypercholesterolemia	22	Across the three groups, for those not receiving treatment, LDL correlated with the percentage of M1 monocytes and M1/M2 ratio. For those receiving treatment a milder correlation between LDL-C and M1/M2 ratio
	Non familial hypercholesterolemia	20	
	Healthy controls	20	
Williams [37]	CVD patients (21) Peripheral arterial disease (10)	31	In controls: ApoA1 correlated with CD163 and inversely with CD86/CD163 on classical monocytes and HDL inversely correlated with CD86/CD163 on classical monocytes
	Healthy controls	33	
Patel [42]	Healthy controls and generally healthy dyslipidemic individuals	30	For all monocyte subsets: HDL-C inversely correlated with IL-1β, CD86, TNFR2, CD319. ApoA1 inversely correlated with IL-1β, TNFR2. Total cholesterol and LDL-C correlated with TLR2, CD163. ApoB correlated with TLR2, CD163, CD93 Other associations for one or two monocyte subsets were observed
Patel [23]	Healthy controls and generally healthy dyslipidemic individuals	30	For all monocyte subsets: ApoA1 inversely correlated with CD62L, CD11b, CD11c and CD29. Other associations (between recruitment markers and ApoA1) for one or two monocyte subsets were observed
***Diet models***			
Gower [63]	High fat feeding of healthy females		*Fasted state*: Monocyte CD11c correlated with Triglycerides, ApoB, total cholesterol/HDL ratio, Non-HDL-C, total cholesterol and LDL-C *After feeding*^5^: Monocyte CD11c expression and arrest on VCAM-1 was elevated—peaking with hypertriglyceridemia, before returning to fasting levels. Monocyte CD11c correlated with triglycerides, Non-HDL-C, ApoB, total cholesterol/HDL ratio, total cholesterol
Short [64]	Male baboons fed a Western diet ^6^	13	*After feeding*: Monocyte chemotaxis and MKP-1 activity correlated with cholesterol

**Notes:** ^1^ Familial hypercholesterolemia: increased plasma cholesterol level, family history, and tendonous xanthoma in patient or first degree relative, polygenic hypercholesterolemia:serum cholesterol > 6.5 mmol/L and no family history of either dyslipidemia or premature CHD, familial combined hyperlipidemia:serum cholesterol > 6.5 mmol/L and serum triglyceride > 2.5 mmol/L and family history of both dyslipidemia and premature heart disease, non-familial combined hyperchol/hypertriglyceridemia:serum cholesterol >6.5 mmol/L and serum triglyceride >2.5 mmol/L without family history of either dyslipidemia or premature CHD. ^2^ Hyperlipidemia: LDL cholesterol >160 mg/dL (>4.14 mmol/L) on two occasions. ^3^ Which group the correlation was in was not mentioned. ^4^ Familial and non-familial hypercholesterolemia categorization was based on the Dutch Lipid Clinical Network criteria. ^5^ Measurements were taken at 3.5 h to match the triglyceride peak and then 7 h when triglcyerides had returned to baseline levels. ^6^ Western diet consisted of high levels of saturated fat, cholesterol and simple sugars. MKP-1 mitogen-activated protein kinase phosphatase 1.

**Table 5:** Monocyte functional changes relative to lipid levels: impact of treatment.

Reference	Clinical Model	N=	Treatment (n)	Monocyte Association
Han [75]	Hypercholesterolemic postmenopausal women	48	Both groups: Estrogen	*At baseline:* Monocyte CCR2 gene expression was significantly elevated in individuals with high LDL-C and low HDL-C, compared to individuals with low LDL-C and low HDL-C. There was a significant correlation between CCR2 gene expression and LDL-C/HDL-C ratios. *Upon treatment:* Estrogen supplement therapy reduced CCR2 gene expression in hypercholesterolemic postmenopausal women but not normocholesterolemic subjects. There was a correlation between the changes in the LDL-C/HDL-C ratio and the changes in monocyte CCR2 gene expression.
	Postmenopausal women (normal cholesterol)			
Okopien [76]	Type 11a dyslipidemia	12	Atorvastatin	*At baseline:* Monocyte IL-1β secretion was elevated in both patient groups compared with the controls. *Upon treatment:* Atorvastatin and Fenofibrate both reduced monocyte IL-1β release and mRNA expression of IL-1β compared to pretreatment.
	Type 11b dyslipidemia	12	Fenofibrate	
	Healthy controls	13		
Okopien [77]	Type 11b dyslipidemia	14	Fenofibrate	*At baseline:* Monocyte IL-1β, IL-6 and Monocyte chemoattractant protein (MCP)-1 levels were higher in hyperlipidemic patients compared to controls. *Upon treatment*: The level of IL-1β, IL-6 and MCP-1 were decreased compared to pre-treatment.
	Healthy controls	12		
Okopien [78]	Atherosclerosis patients with mixed dyslipidemia	52	Atorvastatin, Fenofibrate or Placebo	*At baseline:* Dyslipidemic patients exhibited increased monocyte MCP-1 release compared to control subjects. *Upon treatment:* Atorvastatin and fenofibrate decreased monocyte MCP-1 secretion compared to pretreatment. However, the reduction was not related to the degree of lipid profile improvement.
	Controls ^1^	16		
Okopien [79]	Type IIa dyslipidemic patients	83	Fluvastatin (33), Simvastatin (30) or untreated (20)	*At baseline:* Monocyte secretion of IL-1β was greater from the Type IIa dyslipidemic patients than Type IIb and controls. TNFα release was significantly greater for both patient groups than age matched controls. TNFα release correlated with total cholesterol and ApoB, and IL-1β release positively correlated with total cholesterol, LDL-C and ApoB. *Upon treatment:* Both statins and ciprofibrate reduced monocyte release of TNFα and IL-1β compared with pretreatment. Fenofibrate only reduced TNFα compared with pretreatment. TNFα and IL-1β levels after treatment were not different than controls.
	Type IIb dsylipidemic patients	86	Ciprofibrate (34), Fenofibrate (34) or untreated (18)	
	Controls ^1^	59		
Serrano [80]	Hypercholesterolemia ^2^ with stable coronary artery disease	23	Simvastatin	*At baseline:* Patients’ monocytes had higher levels of CD11b, CD14 and lower L-selectin expression compared with monocytes from control individuals. *Upon treatment*: Statin therapy downregulated CD11b and CD14 whilst increasing L-selectin expression in patients to levels comparable with healthy controls.
	Healthy controls	15		
Krysiak [81]	Impaired glucose tolerance (IGT)	26	Both patient groups: Simvastatin	*At baseline:* Patients’ monocytes released higher levels of TNFα IL-1β, IL-6 and MCP-1 than controls. *Upon treatment:* Simvastatin reduced monocyte cytokine secretion in hypercholesterolemia, but not IGT subjects.
	Hyercholesterolemia ^3^	24		
	Healthy controls	25		
Krysiak [82]	Dyslipidemia ^4^	32	All patient groups: fenofibrate	*At baseline:* Patients’ monocytes produced larger amounts of both IL-1β and MCP-1 than controls. *Upon treatment:* For dyslipidemia (alone) and dyslipidemia with IGT, fenofibrate significantly decreased both IL-1β and MCP-1 but IL-1β was still higher than in controls. For dyslipidemia with IFG, fenofibrate significantly decreased both IL-1β and MCP-1 but both were still higher than in controls.
	Dyslipidemia with impaired fasting glucose (IFG)	32		
	Dyslipidemia with IGT	32		
	Healthy controls	29		
Krysiak [83]	Hypercholesterolemia ^5^	134	Ezetimibe (34),	*At baseline:* Patients’ monocytes released higher levels of TNFα IL-1β, IL-6 and MCP-1 than controls. *Upon treatment:* Compared with placebo, both Simvastatin treatment options reduced monocyte TNFα, IL-1β, IL-6 and MCP-1. For subjects receiving both medications, the levels matched those of healthy controls after 30 days of treatment. Simvastatin alone was superior to ezetimibe in reducing monocyte cytokine release. A longer treatment (90 vs. 30 days) with simvastatin or simvastatin + ezetimibe was more effective in reducing cytokine release.
			Simvastatin (33),	
			Ezetimibe + Simvastatin (35) or	
			Placebo (32)	
	Healthy controls	30		
Krysiak [84]	IFG	28	Both patient groups: Bezafibrate	*At baseline*: IFG and MD subjects had increased monocyte release of MCP-1, IL-6, TNFα and IL-1β. *Upon treatment:* Monocyte release of MCP-1, IL-6, TNFα and IL-1β was normalized in MD subjects, but only MCP-1 and IL-6 normalized in IFG subjects.
	Mixed dyslipidemia (MD) ^6^	29		
	Healthy controls	24		
Dai Perrard [60]	Hypertriglyceridemia ^7^	83	MAT9001 or EPA-EE	*Upon treatment:* Both treatments reduced CD11c and CD36 on CM and IM.
Bekkering [85]	Familial Hypercholesterolemia ^8^	25	Statin	*At baseline:* Patients’ monocytes had higher levels of CCR2, CD11b, CD11c and CD29. They also had a higher production of TNFα, IL-6 and IL-1β *Upon treatment:* CCR2 and CD29 (but not CD11b and CD11c) expression were decreased after statin treatment. Cytokine production remained elevated.
	Healthy controls	20		

**Notes:**^1^ Controls were matched asymptomatic atherosclerotic patients with normal lipid. ^2^ Hypercholesterolemia: LDL-C > 130 mg/dL (>3.36 mmol/L) and fasting triglycerides < 180 mg/dL (<2.03 mmol/L) ^3^ Hypercholesterolemia: total cholesterol >200 mg/dL (>5.17 mmol/L), LDL-C > 130 mg/dL (>3.36 mmol/L), triglycerides < 200 mg/dL (<2.26 mmol/L) ^4^ Dylipidemia: total cholesterol > 200 mg/dL (>5.17 mmol/L), LDL-C > 135 mg/dL (>3.49 mmol/L), triglycerides > 200 mg/dL (>2.25 mmol/L) ^5^ Hyerpcholesterolemia: total cholesterol > 200 mg/dL (>5.17 mmol/L), LDL-C >130 mg/dL (>3.36 mmol/L) and triglycerides < 150 mg/dL (<1.69 mmol/L). ^6^ Mixed dyslipidemia: plasma total cholesterol >200 mg/dL (>5.17 mmol/L), low-density LDL-C >130 mg/dL (>3.36 mmol/L), and triglycerides >200 mg/dL (>2.25 mmol/L). ^7^ Hypertriglyceridemia: triglyceride between 200 and 400 mg/dL (2.25–4.52 mmol/L). MAT9001: Eicosapentaenoic acid (EPA) plus docosapentaenoic acid. EPA-EE: EPA ethyl esters. The study was a crossover design with 14-day treatment of each separated by at least 35 days washout. ^8^ Familial hypercholesterolemia (FH) patients included either definite or probable FH. For all studies, cytokine release is post LPS stimulation.

## Data Availability

Not applicable.
